# Enhancement of the Moisturizing Effect in an Injectable Sodium Hyaluronate Composite Solution by the Addition of Glycine, Alanine, and Proline

**DOI:** 10.1111/jocd.70782

**Published:** 2026-03-06

**Authors:** Anxin Shi, Yayun Guan, Shijie Chen, Yibing Ding, Yingrui Chen, Shuiyun Zeng, Yiqiao Hu, Hong Dong

**Affiliations:** ^1^ State Key Laboratory of Pharmaceutical Biotechnology, Medical School Nanjing University Nanjing China; ^2^ Jiangsu Key Laboratory for Nano Technology Nanjing University Nanjing China; ^3^ Institute of Drug R&D, School of Life Sciences Nanjing University Nanjing China; ^4^ ReaLi Tide Biological Technology (Weihai) Co. Ltd. Weihai China

**Keywords:** HPLC‐MS, injectable sodium hyaluronate composite solution, natural moisturizing factor, skin hydration, vitro evaluation system

## Abstract

**Background:**

Hyaluronate (HA) is widely utilized in skin rejuvenation treatments, yet mono‐component injectable sodium hyaluronate solution (ISHA) is limited by its modest moisturizing performance and frequent dosing requirements. The combination of HA with active ingredients such as amino acids represent a promising strategy to improve hydration efficacy.

**Aims:**

This study aimed to develop a novel injectable sodium hyaluronate composite solution (Co‐ISHA) incorporating three amino acids—glycine, alanine, and proline—and to systematically evaluate its moisturizing effects through phenomenological and mechanistic analyses at cellular and tissue levels.

**Methods:**

This study developed a novel Co‐ISHA containing glycine, alanine, and proline, and evaluated its moisturizing performance through a comprehensive in vitro assessment system. The reparative and protective effects of Co‐HA on cells and tissues under dry conditions were examined, along with the expression of moisturizing‐related genes and natural moisturizing factors (NMF). This system integrated both cellular and tissue‐level models to elucidate the underlying mechanisms.

**Results:**

The results indicated that the addition of these three amino acids significantly enhanced the moisturizing efficacy of hyaluronic acid (HA). Both cellular and tissue‐level evidence confirmed that, compared to single‐component HA, Co‐ISHA more effectively protected and repaired cells damaged by dryness and promoted the production of moisturizing‐related genes and NMF.

**Conclusion:**

This study successfully developed a Co‐ISHA formulation with superior moisturizing properties and established a robust in vitro evaluation system for assessing hydration efficacy. The findings provide a strategic framework for advancing the development of “Cruelty‐Free” cosmetic products.

**Abbreviations**
AQP3: Aquaporin3
CCK‐8: cell counting kit‐8
CD44: CD44 antigen
Cis‐UCA: cis‐urocanic acid
Co‐ISHA: injectable sodium hyaluronate composite solution
COL1A1: collagen type I alpha 1 chain
DMSO: dimethyl sulfoxide
FBS: fatal bovine serun
FLG: filaggrin
HA: hyaluronic acid
HaCaT: human immortalized keratinocytes
HAS: hyaluronan synthase
HPLC: high performance liquid chromatography
HPLC‐MS: high performance liquid chromatography‐mass spectrometry
ISHA: injectable sodium hyaluronate solution
NMF: natural moisturizing factor
PBS: phosphate buffered saline
PCA: l‐pyrrolidone carboxylic acid
ROS: reactive oxygen species
TJP1: tight junction protein 1
Trans‐UCA: trans‐urocanic acid
T‐SKIN: reconstructed human full thickness skin model
UCA: urocanic acid

## 1 Introduction

The skin, as the largest organ of the human body, its water content directly affects the health condition and appearance of the skin. External or internal factors such as skin inflammation, photoaging, and natural aging with age can all lead to a gradual decrease in the main water‐retaining substance, sodium hyaluronate (HA), damage to the skin barrier, and increased water loss, thereby causing dry skin [1]. HA, as a naturally occurring glycosaminoglycan, has become an important component in the field of skin moisturization due to its excellent water‐retaining ability and biocompatibility.

In recent years, sodium hyaluronate injection products have demonstrated significant advantages in skin rejuvenation treatment due to their ability to directly replenish hyaluronic acid in the dermis and repair the skin barrier [2, 3, 4]. However, single‐component sodium hyaluronate injections have issues such as poor moisturizing effect and the need for frequent injections. Studies have shown that combining sodium hyaluronate with other active ingredients, such as amino acids, vitamins, or antioxidants, could significantly enhance its moisturizing effect. However, there is currently a lack of systematic research on the moisturizing efficacy of such composite solutions, and there is also a lack of standardized evaluation systems.

This study aimed to develop a new type of injectable sodium hyaluronate composite solution (Co‐ISHA) and evaluate its moisturizing efficacy through scientific and rigorous methods. The Co‐ISHA developed in this study consists mainly of sodium hyaluronate and supplemented with glycine, alanine, and proline. By directly implanting the aqueous solution of sodium hyaluronate into the dermis, it not only directly replenished water to the dermis but also enhanced the skin's ability to absorb and retain water [5]. A study by Azin Ayatollahi et al. demonstrated that booster therapy with non‐cross‐linked HA is a safe and well‐tolerated procedure and results in improvement of the skin elasticity as well as relative increase in skin hydration [6]. Sodium hyaluronate can form an elastic network structure in the dermis, maintaining the water balance of the skin and mitigating xerosis‐associated symptoms [7]. Additionally, sodium hyaluronate can also form a 3D hydrogel with water, forming a supporting framework between skin cells and enhancing skin elasticity and firmness. Glycine, alanine, and proline serve as nutrients for epidermal keratinocytes and dermal fibroblasts, not only nourishing the cells and maintaining normal physiological functions, but also being the material basis for the synthesis of various moisturizing and barrier repair‐related proteins, such as collagen [8], hyaluronic acid synthase (HAS1‐3) [9], CD44 [10], aquaporin (AQP3) [11] and so on.

During the development of cosmetics, pharmaceuticals, and related products, efficacy and safety assessments are conducted to determine product performance, encompassing both preclinical and clinical evaluations. Preclinical evaluations often include animal testing and in vitro alternative testing methods. Animal studies have played a crucial role in analyzing the safety and efficacy of cosmetics, assessing the exposure risks of chemical components, and facilitating the development and testing of cosmetics, drugs, vaccines, and other products [12]. However, growing concerns over animal welfare have prompted some countries and brands to promote certification systems for “Cruelty‐Free” cosmetics. The certification criteria typically require that product ingredients contain no animal‐derived components and that the final product has not been tested on animals [13]. Examples of such certifications include the “Leaping Bunny” program in Europe and North America, Australia's “Choose Cruelty Free” organization, and the “People for the Ethical Treatment of Animals” (PETA) in the United States. In line with this global trend, China also eliminated mandatory animal testing for imported general cosmetics in 2021.

Despite these advancements, some scientists maintain that animal testing remains essential in the cosmetics field, arguing that it is as critical as alternative methods for ensuring product safety. Conversely, opponents contend that the scientific conclusions drawn from animal experiments in cosmetics are unreliable, citing issues with reproducibility, reliability, and relevance [14]. Driven by these divergent viewpoints, the development of robust alternative testing methods for cosmetics has become increasingly vital.

The in vitro evaluation method developed in this study, which used immortalized human keratinocytes (Haacht) cells and human full‐thickness skin models (T‐SKIN), evaluated the moisturizing efficacy of the Co‐ISHA at both the cellular and tissue levels, and explored its moisturizing mechanism. This method not only enabled efficient and convenient evaluation of the product's effectiveness during the development of Co‐ISHA products, but also reduced the reliance on animal experiments in the development process.

## 2 Materials and Methods

### 2.1 Experimental Samples

The hyaluronic acid used in the injectable sodium hyaluronate solution (ISHA) and Co‐ISHA for this study was non‐cross‐linked and had a molecular weight ranging from 700 kD to 1700 kDa. Glycine, Alanine, and Proline (Hubei Bafeng Pharmaceutical Co. Ltd.) were incorporated into the Co‐ISHA formulation.

### 2.2 Cell Culture

HaCaT cells (Shanghai Mirror Quantum Cell Technology Co. Ltd.) were cultured in DMEM supplemented with 10% FBS at 37°C and 5% CO_2_. Log‐phase cells were seeded into 96‐well plates at 1 × 10^4^ cells/well and incubated for 24 h before treatment.

### 2.3 Cell Dryness‐Injury Experiment

Groups: Blank Control (Control), Negative Control (Dry), Positive Control (Dry + glycerol) Co‐ISHA (Dry + Co‐ISHA), and ISHA (Dry + ISHA). Each group contained six replicate wells (100 μL/well).

Dryness conditions: The culture medium was removed in a biosafety cabinet, and cells were air‐dried at 20°C–25°C with airflow of 0.32–0.40 m/s for 10 min.

#### 2.3.1 Concentration Optimization (Test Samples and Positive Control)

Glycerol and Co‐ISHA were serially diluted in culture medium. Final glycerol concentrations were 20, 10, 5, 2.5, and 1.25 mg/mL; Co‐ISHA dilution factors were 2, 5, 10, and 50. After 24 h treatment, dryness injury was induced and cell viability was measured by CCK‐8. Concentrations for subsequent experiments were selected relative to the negative control.

#### 2.3.2 Protection During Dryness Injury

Per the group assignments, solutions were added and cells were incubated for 24 h prior to dryness injury (except the Blank Control). After injury, cell viability was assessed by CCK‐8.

#### 2.3.3 Recovery After Sryness Injury

All groups except the Blank Control underwent dryness injury. Group solutions were then added and incubated for 24 h, followed by CCK‐8.

### 2.4 Cell DMSO‐Injury Experiment

Groups: Blank Control (Control), Negative Control (DMSO) Co‐ISHA (DMSO + Co‐ISHA), and ISHA (DMSO + ISHA); six replicate wells per group (100 μL/well).

#### 2.4.1 Injury‐Time Optimization

HaCaT cells were exposed to DMEM containing 10% DMSO for 0.5, 1, 2, 3, 4, 5, or 6 h before CCK‐8. Untreated cells served as the blank control.

#### 2.4.2 Concentration Optimization (Test Samples)

Co‐ISHA was diluted 2×, 5×, 10×, and 50× in culture medium. Cells were incubated with the dilutions for 23 h, the medium was removed, and fresh medium containing 10% DMSO was added (except Blank Control). The Blank Control received medium without DMSO. After 1 h at 37°C/5% CO_2_, cell viability was measured. An appropriate dilution factor was selected for subsequent experiments.

#### 2.4.3 Recovery After DMSO Injury

Groups were as above. Except for the Control, all groups were treated with medium containing 10% DMSO for 2 h, washed once with PBS, and then incubated for 24 h with either (i) medium containing 50% PBS (Control and DMSO) or (ii) medium containing 50% of the respective stock solution (DMSO + Co‐ISHA, DMSO + ISHA). Cell viability was then measured.

#### 2.4.4 Protection During DMSO Injury

The Control and DMSO groups received medium containing 50% PBS; the DMSO + Co‐ISHA and DMSO + ISHA groups received medium containing 50% of the respective stock solution. After 22 h, the medium was removed. All groups except Control were then exposed to medium containing 10% DMSO for 2 h, washed once with PBS, and subjected to the cell‐viability assay.

### 2.5 Evaluation of Intracellular ROS

Log‐phase HaCaT cells were seeded into 12‐well plates at 2 × 10^5^ cells/well and cultured for 24 h at 37°C. Groups: Control (medium + 50% PBS), DMSO (medium + 50% PBS + 1% DMSO), DMSO + Co‐ISHA (medium + 50% Co‐ISHA stock + 1% DMSO), DMSO + ISHA (medium + 25% ISHA stock + 1% DMSO). Each group had three replicates and was cultured for 24 h. After washing three times with sterile PBS, DCFH‐DA was diluted to 5 μmol/L in PBS and 1 mL was added per well. Plates were incubated for 30 min at 37°C.

#### 2.5.1 Flow Cytometry

Following incubation, the probe solution was removed, cells were washed once with PBS, digested with trypsin (300 μL, 3 min, 37°C), neutralized with 600 μL medium, collected, and centrifuged (2000 rpm, 3 min). Pellets were resuspended in 300 μL PBS and analyzed for FITC fluorescence by flow cytometry.

#### 2.5.2 Confocal Microscopy

After probe incubation, cells were washed three times with PBS and imaged by confocal laser scanning microscopy (CLSM) to visualize DCF fluorescence.

### 2.6 qRT‐PCR for Moisturization‐Related Genes

Log‐phase HaCaT cells were seeded in 6‐well plates at 2 × 10^5^ cells/well and cultured for 24 h at 37°C. Groups: Control Co‐ISHA (2.5 mg/L), ISHA (2.5 mg/L). After 24 h treatment, RNA was extracted, reverse‐transcribed to cDNA, and qPCR was performed using SYBR Green. GAPDH served as the reference gene, and relative expression was calculated by the 2^−ΔΔCt^ method.

The forward primer (5′→3′) sequences:

*AQP3*: TCAAGCTGCCCATCTACACC;

*CD44*: CTGCCGCTTTGCAGGTGTA;

*COL1A1*: GCCCTGTTGGTGTTCAAGGA;

*FLG*: GGACAGGAACAATCATCGGGG;

*HAS1*: GAAGTTCCTGGGTACCCAC;

*HAS2*: CTCGCAACACGTAACGCAAT:

*HAS3*: GGAAGGTTTTGCTGCCTTGG;

*TJP1*: ACCAGTAAGTCGTCCTGATCC.

The reverse primer (5′→3′) sequences:

*AQP3*: GTCAACAATGGCCAGCACAC;

*CD44*: CATTGTGGGCAAGGTGCTATT;

*COL1A1*: TTCCAGTCAGACCCTTGCA;

*FLG*: CAACCTCTCGGAGTCGTCTG;

*HAS1*: GGAGGTGTAVTTGGTAGCA;

*HAS2*: AGTGTCTGAATCACAAACCTGT;

*HAS3*: GCACCGGCATCCTGCAA;

*TJP1*: TCGGCCAAATCTTCTCACTCC.

### 2.7 Extraction and Quantification of Natural Moisturizing Factor (NMF)

#### 2.7.1 HaCaT Cell Treatment and NMF Extraction

HaCaT cells were seeded at a density of 4 × 105 cells/well in a 6‐well plate, with 2 mL of culture medium added to each well and cultured at 37°C for 24 h. After the cells adhered to the wall, DMEM medium was added to the control group Co ISHA diluted twice with DMEM medium was added to the Co ISHA treatment group, and ISHA diluted four times with DMEM medium was added to the ISHA treatment group. Three replicates were set up in each group and cultured for 24 h. The cells were washed once with PBS, digested with trypsin, collected after digestion, centrifuged at 1000 r/min for 3 min, and discarded from the culture medium. Add 250 μL of methanol to each sample, sonicate for 30 min, centrifuge at 14 000 r/min for 10 min at 4°C. Transfer the supernatant to a new centrifuge tube and dry it with a nitrogen blower; Add 250 μL of deionized water and sonicate for 30 min; Centrifuge at 4°C for 10 min at a speed of 12 000 r/min; Suck 194 μL of supernatant into the liner tube, add 5 μL of 1M ammonium formate mother liquor and 2.5 μL of acetonitrile, shake well, and wait for machine operation.

#### 2.7.2 T‐SKIN Treatment and NMF Extraction

The Control received PBS; the Co‐ISHA group received stock solution; the ISHA group received stock solution diluted 2× in PBS. Each group had three replicates. Using a sterile mesotherapy needle, 50 μL of each sample was injected into the dermis at three sites and cultured for 24 h. The model surface was washed 15 times with sterile PBS. Tissue of uniform size was excised with a mold and transferred to centrifuge tubes. Proteinase K working solution (0.2 mg/mL; 500 μL) was added for enzymatic hydrolysis (50°C, 1 h). Tissue was then transferred to new tubes and 250 μL methanol was added. Samples were sonicated 30 min and centrifuged (14 000 rpm, 10 min, 4°C). Supernatants were dried under nitrogen, reconstituted in 250 μL deionized water, sonicated 30 min, centrifuged (12 000 rpm, 10 min, 4°C), and 194 μL supernatant was transferred to a liner tube. Then 5 μL 1M ammonium formate and 2.5 μL acetonitrile were added and mixed before injection.

#### 2.7.3 HPLC–MS Conditions

Column: C18, 5 μm, 4.6 × 250 mm; mobile phase: 20 mmol/L ammonium formate (990 mL) with acetonitrile (10 mL), filtered through a 0.45 μm membrane and sonicated 15 min; column temperature: 30°C; flow rate: 0.3 mL/min; injection volume: 20 μL; detection wavelengths: 270 nm (cis‐UCA, trans‐UCA) and 210 nm (PCA); molecular weights: 138.12 (cis‐UCA, trans‐UCA), 129.11 (PCA); retention times: 1.08 min (cis‐UCA), 1.60 min (trans‐UCA), 2.88 min (PCA).

#### 2.7.4 Standard Curves

PCA, cis‐UCA, and trans‐UCA standards were prepared and analyzed as in Section 2.7.3 to generate standard curves. Stock solutions were prepared at 8 mg/mL (PCA), 5 mg/mL (cis‐UCA), and 0.5 mg/mL (trans‐UCA); serial dilutions produced the following gradients: PCA, 256, 64, 16, 4, 1, 0.5 ng/mL; cis‐UCA, 128, 32, 8, 2, 0.5, 0.125 ng/mL; trans‐UCA, 5.12, 1.28, 0.32, 0.08, 0.02, 0.005 ng/mL.

#### 2.7.5 Quantification

Analytes were quantified under the conditions in Section 2.7.3. PCA and UCA contents were calculated from peak areas using the standard curves.

#### 2.7.6 Calculations

Total content of each analyte = measured concentration × total volume; total UCA = cis‐UCA + trans‐UCA.

### 2.8 Data Processing and Analysis

Data were analyzed with GraphPad Prism 9.2. For two‐group comparisons, Student's *t*‐test was used; ANOVA was used for multiple groups. *p* < 0.05 was considered statistically significant.

## 3 Results

### 3.1 Co‐ISHA Repairs Dryness‐Induced Damage in HaCaT Cells

Dryness damages skin cells by increasing oxidative stress and generating free radicals that disrupt membrane structure and the cytoskeleton, reduce viability, and promote apoptosis [15]. Prolonged exposure accelerates skin aging, causing wrinkles and laxity. We therefore evaluated whether Co‐ISHA protects against and repairs dryness‐induced cell damage.

To evaluate the protective and reparative effects of the Co‐ISHA, this study utilized a desiccation injury model on human immortalized keratinocytes (HaCaT cells). HaCaT cells were dried for 10 min at room temperature (20°C–25°C) in a laminar flow hood with an airflow velocity of 0.32–0.4 m/s. We designed two experimental protocols: post‐injury recovery experiment and protection experiment. In the post‐injury recovery experiment, the test substance was added to initiate repair after the cells had undergone drying‐induced damage. In the protection experiment, cells were cultured with a medium containing the test substance before being subjected to drying‐induced damage.

**FIGURE 1:**
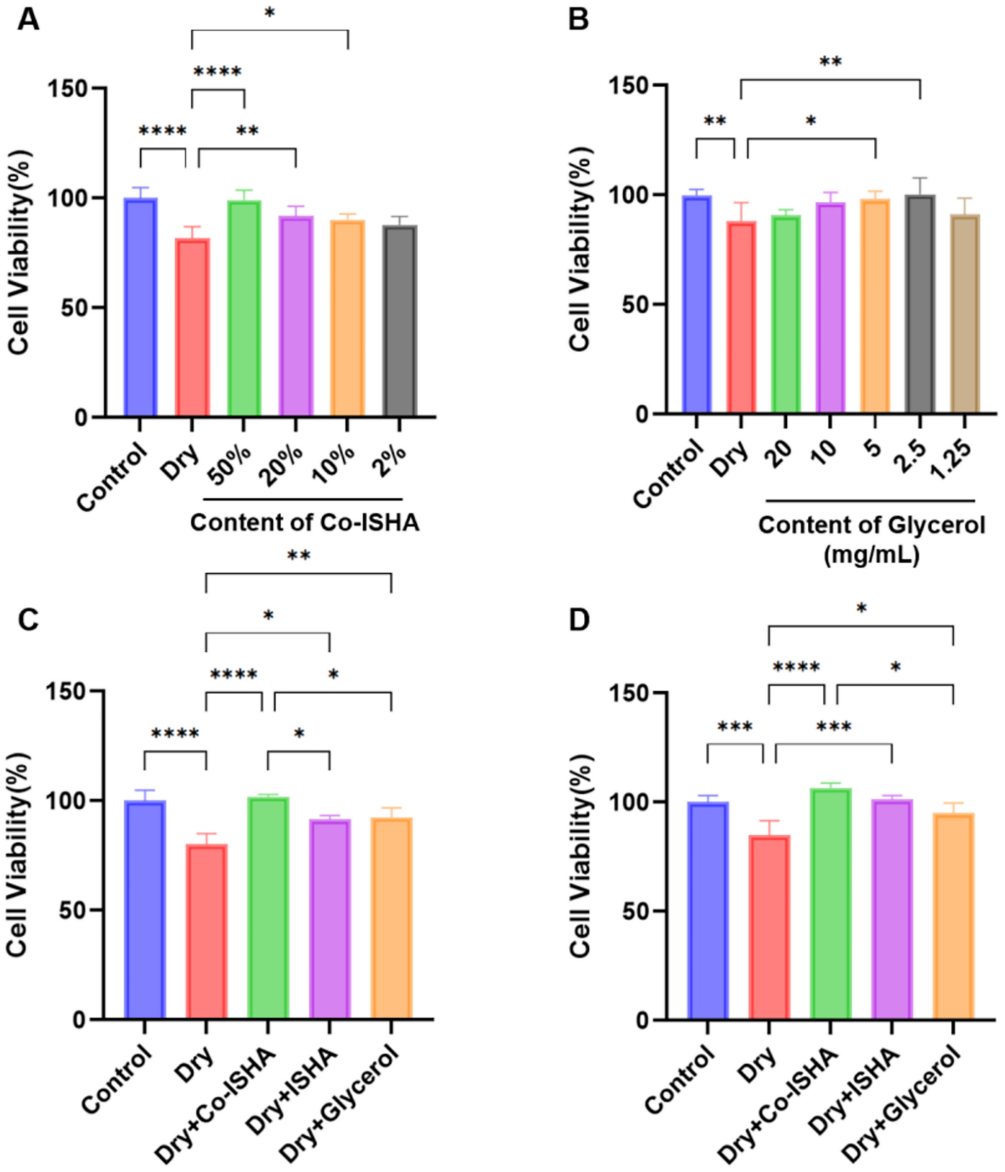
Co‐ISHA protected and repaired dry‐damaged HaCaT cells ($\bar{x}$ ± *s*, *n* = 6, ****p < 0.0001, ***p < 0.001, **p < 0.01, *p < 0.05). (A) Exploration of Co‐ISHA administration dose. (B) Exploration of glycerol administration dose. (C) Experiment on repair after dry injury. (D) Experiment on protection during dry injury.

Glycerol, known for its excellent moisturizing properties and safety profile, is widely used in various skincare and personal care products. In this study, glycerol was employed as a positive control to assess the moisturizing capacity of Co‐ISHA [16]. Although high concentrations of glycerol provide effective moisturization, excessively high levels can exert toxic effects on mammalian cells and inhibit cell proliferation [17]. Therefore, we first optimized the dosing concentrations for Co‐ISHA and glycerol. We indentified that 50% Co‐ISHA and 2.5 mg/mL glycerol were the optimal concentrations (Figure 1A,B). The HA concentration in ISHA was twice that in Co‐ISHA; therefore, 25% ISHA was used for comparability. In post‐injury recovery experiments, both Co‐ISHA and ISHA significantly increased viability relative to Control, with Co‐ISHA outperforming ISHA and glycerol (Figure 1C). In protection experiments, pretreatment with Co‐ISHA or ISHA maintained significantly higher viability after injury, and Co‐ISHA again showed the greatest effect (Figure 1D). These findings indicate that Co‐ISHA both repairs and protects against dryness‐induced damage.

### 3.2 Co‐ISHA Repairs Chemical Injury Induced by DMSO

DMSO is highly permeable; rapid exposure can dehydrate cells, mimicking osmotic imbalance and producing dry‐like damage [18, 19]. We used 10% DMSO to model chemical injury and assessed repair/protection by ISHA and Co‐ISHA via CCK‐8.

**FIGURE 2:**
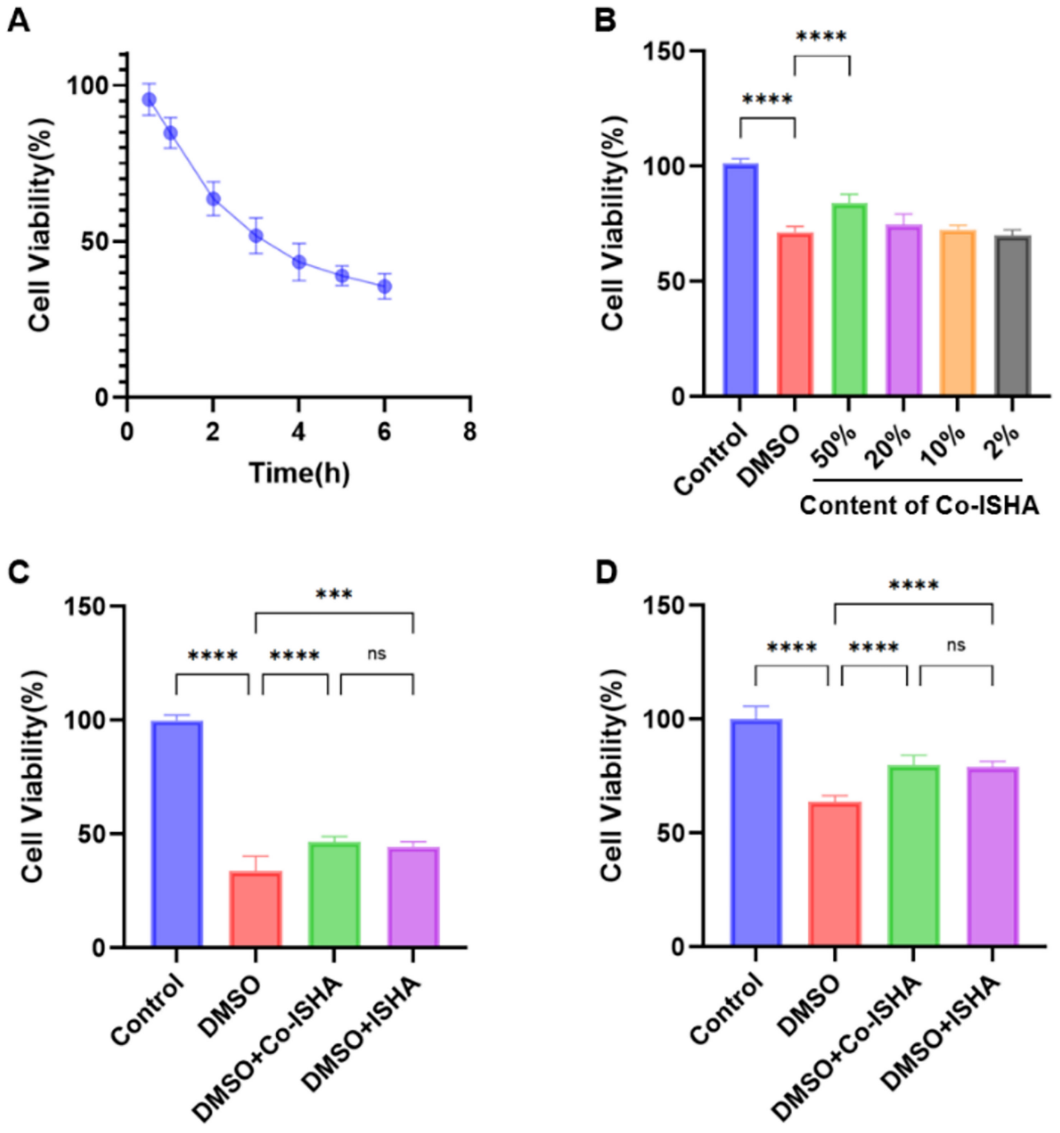
Co‐ISHA protected and repaired cells damaged by DMSO ($\bar{x}$ ± *s*, *n* = 6, ****p < 0.0001, ***p < 0.001, ns: no significance). (A) Exploration of DMSO damage duration. (B) Exploration of Co‐ISHA administration dose. (C) Experiment on repair after DMSO damage. (D) Experiment on protection during DMSO damage.

Firstly, we used a culture medium containing 10% DMSO to cause cell damage for different durations, in order to determine the subsequent DMSO damage time (Figure 2A). After DMSO treatment, the viability of HaCaT cells significantly decreased, indicating that the cell viability of HaCaT was inhibited under the action of DMSO drying damage. Therefore, we selected the 2‐h treatment group with a cell survival rate of 63.6% for the subsequent experiments. Next, we screened an appropriate concentration of Co‐ISHA. 50% Co‐ISHA could significantly increase the survival rate of HaCaT cells in the post‐injury recovery experiment (Figure 2B). Hence, subsequent experiments were conducted using 50% Co‐ISHA and the same concentration of ISHA. In this section, we also conducted post‐injury recovery experiment (Figure 2C) and protection experiment (Figure 2D). Compared with the negative control group, both Co‐ISHA and ISHA were able to repair and protect cells from DMSO‐induced damage.

### 3.3 Co‐ISHA Reduces Intracellular ROS

**FIGURE 3:**
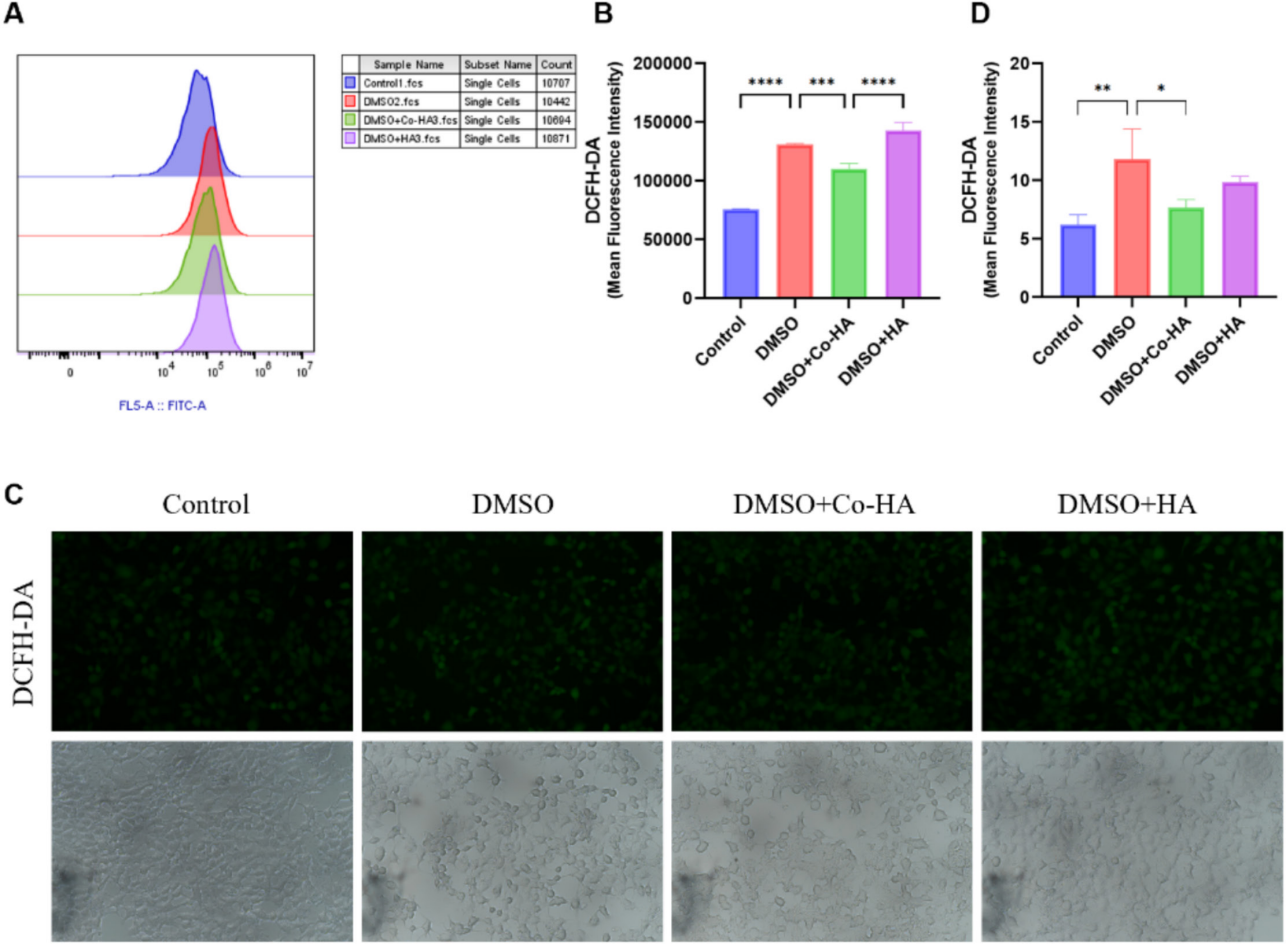
Co‐ISHA removed ROS from cells damaged by DMSO ($\bar{x}$ ± *s*, *n* = 3, ****p < 0.0001, ***p < 0.001, **p < 0.01, *p < 0.05). (A, B) Flow cytometry was used to measure the fluorescence intensity of DCFH‐DA in the cells. (C, D) CLSM was employed to detect the fluorescence intensity of DCFH‐DA in the cells.

Environmental damage often acts through oxidative stress. Oxidative stress damages the skin's defense by rapidly depleting the antioxidant capabilities of enzymes (glutathione peroxidase, glutathione reductase, superoxide dismutase, and catalase) and non‐enzymes (vitamin E, vitamin C, and glutathione), thereby leading to harmful effects [20]. Dryness or chemical insult increases intracellular ROS, resulting in skin aging and damage. The DCFH‐DA fluorescent probe is a commonly used method for detecting the content of ROS in cells. The non‐fluorescent DCFH‐DA has good cell membrane permeability and undergoes an esterase hydrolysis reaction within the cell, generating the product DCFH that has no fluorescence and cannot pass through the cell membrane. However, under the action of intracellular ROS, the non‐fluorescent DCFH is oxidized to the fluorescent DCF. The stronger the fluorescence intensity, the greater the content of DCF generated, and the higher the level of ROS within the cell [21]. We quantified the level of intracellular ROS by flow cytometry (Figure 3A,B) and CLSM (Confocal Laser Scanning Microscope) (Figure 3C,D). After 24 h with 1% DMSO, DCF fluorescence was significantly increased, confirming the oxidative‐stress model. Compared with the DMSO group and the DMSO + ISHA group, the fluorescence intensity of the DMSO + Co‐ISHA group significantly decreased, indicating that Co‐ISHA had a very positive effect in removing intracellular ROS.

### 3.4 Co‐ISHA Upregulates Moisturization‐Related Genes in HaCaT Cells

Skin hydration is maintained primarily by NMF, the lipid barrier, and water‐channel proteins. Multiple genes play important roles in skin moisturization. AQP3 (Aquaporin3) is a water/glycerol transporter expressed in epidermal keratinocytes, and the level of epid ermal glycerol relates to the water cooperation of the stratum corneum [22]. The water and glycerol transport mediated by AQP3 involves the migration and proliferation of keratinocytes, and plays an important role in skin repair [23, 24]. NMF is an important component of the skin stratum corneum, mainly composed of products of silk proteoglycans, and its content reduction is closely related to skin dryness and other problems. NMF maintains the skin hydration state and barrier function through moisture absorption and water retention [25]. NMF is derived largely from filaggrin (FLG). FLG gene mutation is closely related to atopic dermatitis and ichthyosis, leading to skin barrier function defects and water loss [26, 27, 28]. HAS (Hyaluronan Synthase) is an enzyme responsible for synthesizing HA. In mammals, there are three homologous genes encoding related proteins, HAS1‐3 [29]. HAS1 and HAS3 highly express in the skin, respectively synthesizing medium and low molecular weight HA. HAS2 mainly synthesizes high molecular weight HA, which highly expresses in joint synovial cells. The HAS protein family is involved in various processes such as extracellular matrix formation and maintenance [10, 30]. CD44 is a ubiquitous cell surface receptor that can promote keratinocyte activity after binding to HA, improving epidermal function [3]. A reduction in endogenous hyaluronic acid (HA) within keratinocytes was observed in CD44 knockout mice, leading to the loss of certain functions and causing changes in various physicochemical indicators of the skin, as well as alterations in barrier function [2]. TJP1 (Tight Junction Protein 1) is a core scaffold protein of tight junctions, which maintains cell polarity through interaction with various proteins, ensures the separation of the cell apex and base sides, regulates the integrity of the tight junction structure to form a selective barrier, and controls paracellular permeability [31, 32, 33]. COL1A1 (Collagen Type I Alpha 1Chain) encodes type I collagen, a major component of the extracellular matrix of dermal cells. The low level of type I collagen in senescent skin fibroblasts links to the transcriptional inhibition of COL1A1 [34]. Maintaining the level of fibronectin collagen in the skin plays a role in anti‐aging [34].

**FIGURE 4:**
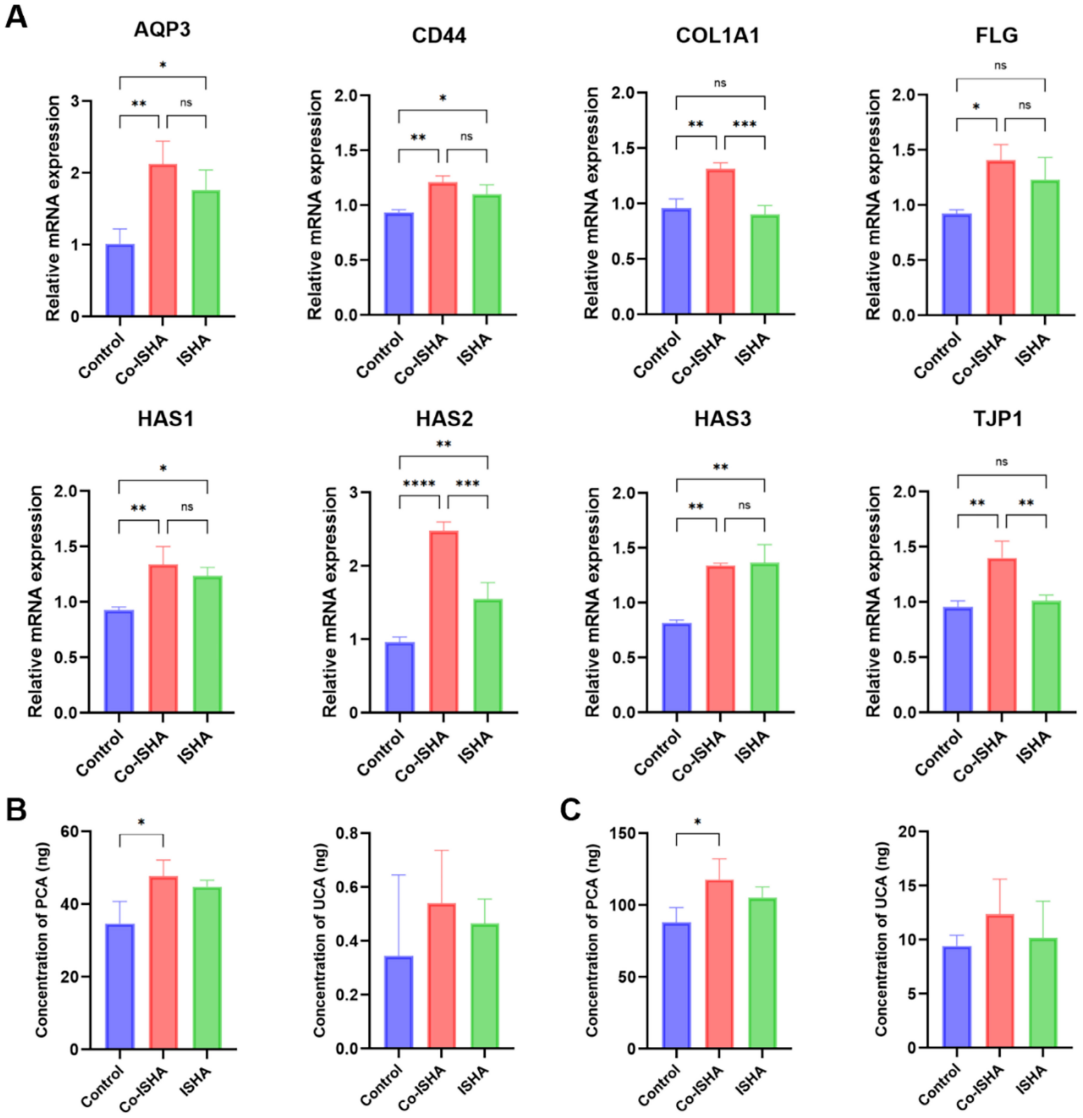
Co‐ISHA promoted the expression of genes related to moisture retention and increases the content of NMF in tissues and cells ($\bar{x}$ ± *s*, *n* = 4, ****p < 0.0001, ***p < 0.001, **p < 0.01, *p < 0.05, ns: no significance). (A) The mRNA transcription levels of *AQP3*, *CD44*, *COL1A1*, *FLG*, *HAS1*, *HAS2*, *HAS3*, and *TJP1* in HaCaT cells after Co‐ISHA treatment; (B) The content of NMF in HaCaT cells after Co‐ISHA treatment; (C) The content of NMF in the human full‐thickness skin model after Co‐ISHA treatment.

In order to explore the mechanism by which Co‐ISHA exerts its skin moisturizing effect, this study used the same concentration of Co‐ISHA and ISHA to act on HaCaT cells for 24 h. After collecting the cells, RNA was extracted and reverse transcribed into cDNA, and q‐PCR experiments were conducted to analyze the expression levels of various moisturizing‐related genes in HaCaT cells (Figure 4A). qPCR revealed that Co‐ISHA significantly increased AQP3, COL1A1, HAS2, and TJP1 expression compared with Control and ISHA (Figure 4A). CD44, FLG, HAS1, and HAS3 were significantly higher than Control and showed modest improvement versus ISHA. These results suggest that Co‐ISHA promotes NMF production and endogenous HA synthesis, supports collagen synthesis, helps maintain water–glycerol balance, and strengthens the barrier.

### 3.5 Co‐ISHA Increases NMF in HaCaT Cells and a 3D Skin Model

The epidermis, the outermost layer of the skin, is primarily composed of keratinocytes. It protects the body from harm caused by the external environment, such as preventing water loss, blocking ultraviolet radiation, and resisting microbial invasion [35]. The outermost layer of the epidermis is the stratum corneum (SC), its structure and composition are pivotal for hydration; damage leads to dryness [35].

The introduction of osmotically active substances—small polar compounds that reduce the chemical potential of water—can protect the stratum corneum from severe desiccation. Healthy skin is rich in such osmotically active compounds, known as NMF [36]. The NMF mixture accounts for up to 10% of the dry weight of keratinocytes, including various amino acids and their derivatives, such as uric acid (UCA), pyrrolidone carboxylic acid (PCA), as well as lactic acid, sugars, urea, glycerol, etc. [37, 38]. Winter dryness, atopic dermatitis, and skin dryness are associated with reduced NMF levels [39, 40].

Mesotherapy‐type HA injections deliver solution to the dermis to moisturize skin [41]. Whether such treatment increases NMF is an important efficacy marker. Because FLG degradation is a major NMF source, and Co‐ISHA increased FLG expression (Section 3.4), we quantified PCA and UCA in HaCaT cells and in a reconstructed full‐thickness skin model (T‐SKIN) by HPLC–MS. This was done to validate the ability of Co‐ISHA to promote NMF expression at both cellular and tissue levels. In T‐SKIN, fibroblasts form the dermis and normal human keratinocytes form a stratified epidermis. Co‐ISHA or ISHA was injected into the dermis; after 24 h, NMF was extracted and PCA/UCA measured. In cells Co‐ISHA or ISHA was added for 24 h before NMF extraction. In both systems Co‐ISHA increased PCA and UCA versus Control and ISHA, with PCA showing a significant increase over Control (Figure 4B,C).

In summary Co‐ISHA promotes NMF production at the cellular and tissue levels, consistent with improved moisturization.

## 4 Discussion

Skin moisturization is a crucial factor for maintaining the skin's barrier function and health. As people's demand for facial rejuvenation becomes increasingly higher, hyaluronic acid injections have gained significant attention in the field of skin moisturization. This study developed a topical sodium hyaluronate composite solution and established a reliable in vitro pharmacodynamic evaluation system. Skin moisturization is mainly maintained by NMF, lipid barrier, and water channel proteins. Gene regulation played a central role in these processes, and mutations or abnormal expressions of related genes may lead to skin dryness, impaired barrier function, and related diseases. This study evaluated the moisturizing effect of Co‐ISHA comprehensively through the construction of two models of cells and tissues, and explored its possible molecular mechanisms.

The results showed that Co‐ISHA could effectively protect HaCaT cells from drying and DMSO damage, as well as repaired cellular damage, which proved that Co‐ISHA had a good moisturizing effect. To explore its possible mechanism, we first evaluated its ability to remove ROS. We found that ROS production in cells treated with Co‐ISHA was significantly reduced, demonstrating that Co‐ISHA might protect and repair cells by eliminating ROS. In addition, we conducted qPCR experiments, and found that the expression of eight moisturizing‐related genes in HaCaT cells treated with Co‐ISHA was significantly increased, suggesting that Co‐ISHA played an important role in promoting the synthesis of NMF, endogenous ISHA, collagen, and maintaining the water‐glycerol balance, as well as improving the skin barrier and other gene pathways. It was worth noting that the added compound (glycine, alanine, and proline) played an important role in promoting collagen synthesis and improving the skin barrier in these two pathways. Finally, we detected the effect of Co‐ISHA on NMF at the cellular and tissue levels. We found that Co‐ISHA could significantly increase the content of PCA, the main NMF, and also enhance the content of another main NMF, UCA.

In conclusion, as a Co‐ISHA featured hyaluronic acid sodium and other added substances (glycine, alanine, and proline) that work together to eliminate ROS in damaged skin cells, promote the expression of moisturizing‐related genes, increase the NMF content in the skin, and thereby achieve a good moisturizing effect.

## Author Contributions

Anxin Shi contributed to the study conception, design, data collection, and analysis. Yayun Guan contributed to material preparation, data collection and analysis. Shijie Chen contributed to material preparation and data collection. Yibing Ding made contributions to experimental techniques and analysis. Yingrui Chen contributed the key research materials, HA and Co‐HA, for this study. This study was completed under the guidance of Hong Dong, Yiqiao Hu and Shuiyun Zeng. The first draft of the manuscript was written by Anxin Shi and Yayun Guan, all authors commented on previous versions of the manuscript. All authors read and approved the final manuscript.

## Funding

This study was supported by National Natural Science Foundation of China (Grant 82241054) and Collaborative Innovation Project of Yangtze River Delta Science and Technology Community (Grant 2023CSJZN0800).

## Ethics Statement

All experiments conducted in this study were performed in vitro and did not involve any animal or human experiments. All cell lines and tissue models used in this study were commercial products purchased from reputable suppliers. The human immortalized keratinocytes (Hacat) cells were purchased from iCell Bioscience Inc. (Shanghai, China). T‐Skin were purchased from EPISKIN (Shanghai, China). The acquisition and use of these products comply with ethical standards, and all necessary ethical reviews and informed consent procedures for their original sources have been completed by the suppliers. Biological waste generated during the experiments was disposed of in accordance with laboratory biosafety management regulations.

## Consent

The human cell lines and tissue models used in this study are commercially available products purchased from iCell Bioscience Inc. (Shanghai, China) and EPISKIN (Shanghai, China). The suppliers have ensured the compliance of the original collection and establishment processes for these products, obtaining all necessary ethical approvals and donor informed consent. Therefore, this study does not raise any new ethical concerns.

## Conflicts of Interest

The authors declare no conflicts of interest.

## Data Availability

The data that support the findings of this study are available from the corresponding author upon reasonable request.
